#  Ileocecal patch –low rectal anastomosis in total colectomy: New idea for the prevention of fecal incontinence

**Published:** 2014

**Authors:** Valiullah Mehrabi, Leily Mohajerzadeh, Alireza Mirshemirani, Ahmad Khaleghnejad Tabari, Azadeh Falahi, Shabnam Abtahi, Marjan Kafaei

**Affiliations:** 1 Department of Pediatric Surgery, Pediatric Medical Center, Tehran University of Medical Sciences, Tehran, Iran.; 2 Pediatric Surgery Research Center, Shahid Beheshti University of Medical Sciences, Tehran, Iran.

**Keywords:** Total colostomy, Ileocecal patch, Ulcerative colitis, Hirschsprung.

## Abstract

***Background: ***Total colectomy is used in children with total colonic aganglionosis, Ulcerative colitis (UC) and familial adenomatous polyposis (FAP). The purpose of this study was to maintain ileocecal valve and rectal-sparing surgery for the prevention of fecal incontinence in these children.

***Methods:*** From1990 to 2011, 14 children with diagnosis of UC, FAP and Hirschsprung’s disease were operated. Total colectomy was done with the preservation of patch of cecum with ileocecal valve and half of the rectum with ileocecorectal anastomosis. Distal ileum designed as S shape pouch and ileocecal valve were preserved. In Hirschsprung’s disease, posterior rectal myotomy was established. The data were collected and analyzed.

***Results:*** The mean age of the patients was 54 months (ranged from 2 months to 18 years). Ten patients were male. Among 14 patients, Hirschsprung’s disease, ulcerative colitis and FAP were seen in 10, 3, and one case, respectively. They were followed up annually. Clinical and endoscopic examinations were performed to evaluate the function of ileocecorectal anastomosis. They followed from 2 to 24 years. At first year, the patients experienced four to six bowel movements during the day and one at night. This frequency decreased over time. The main postoperative complications included recurrent enterocolitis (n=2), perianal fistula (n=2). Only 2 patients were suffering from some degree of fecal soiling.

***Conclusion: ***The results show that the Ileocecal patch- low rectal anastomosis in total colectomy leads to low complications and prevent fecal frequency and incontinence. It also increases absorptive function of ileum in children.

Most centers originally employed the straight pull-through technique for patients with ulcerative colitis and familial polyposis or Hirschsprung's disease (1). Coran AG(2) in 1981, used endorectal pull-through for the management of ulcerative colitis. There are different surgical strategies in management of FAP and ulcerative colitis. Some advocate to colectomy and ileocecal anastomosis and others recommend protectomy and ileal pouch anal anastomosis (IPAA) (3). Total proctocolectomy is definitive surgical procedure for the management of ulcerative colitis, total colonic agangliosis and familial adenomatous polyposis. There are some techniques for total proctocolectomy either open or laparoscopic (4), but these procedures are very aggressive and have many complications such as neurogenic bladder, pelvic abscess, and sexual dysfunction.

In our study, the preservation rectum and ileocecal valve with use of reservoirs, stool frequency decreased. Rectum-sparing surgery is occasionally recommended, so there is no pelvic manipulation and leads to the improvement of quality of life, with pelvic innervations, and sexual function salvage (1). There are many ways to improve the absorptive function of ileum and reduce bowel movement such as Duhamel-Martin procedure and ileoanal patch (5). Maintaining ileocecal valve and rectum-sparing surgery can prevent fecal incontinence and improve the quality of life in these children with less pelvic dissection and the maintenance of two basic valves of GI.

## Methods

Between July 1990 and October 2011, 14 children (3 with ulcerative colitis, one with familial polyposis, and 10 with Hirschsprung’s disease) underwent total colectomy and ileocecorectal anastomosis without a back-up ileostomy. 

**Figure 1:**
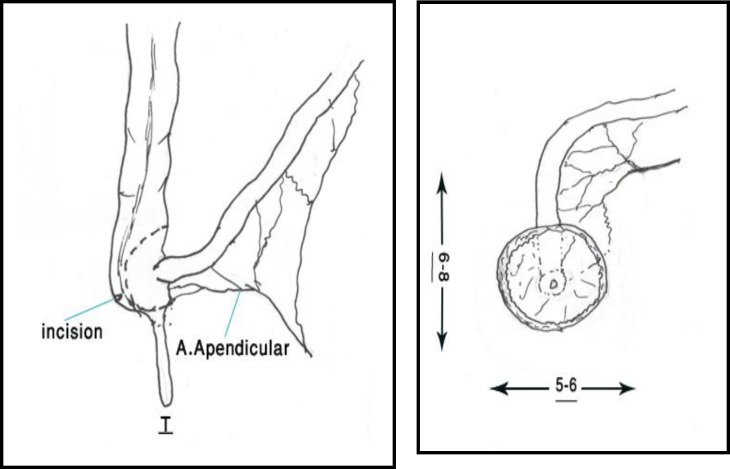
Resection of 20 centimeters of terminal ilium including iliocecal valve and part of cecum

**Figure 2:**
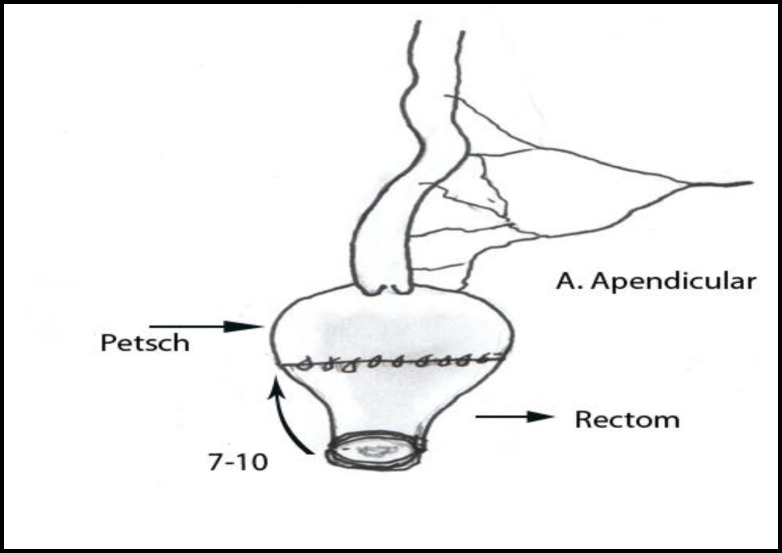
Anastomosis of terminal ilium patch as shown above to the rectum

We presumed that by preserving two important valves of gastrointestinal tract that include ileocecal and rectum can prevent incontinence and prolong bowel transit, thus improve the intestinal absorption. We selected our cases from patients who needed total colectomy. Consequently they were patients with total colonic agangliosis with intact ileum, ulcerative colitis without rectal involvement and familial adenomatous polyposis without polyps in rectum. Before surgery all patients underwent barium enema, colonoscopy and biopsy. In hirschsprung disease cases, we sent multiple specimens for frozen section. We performed total colectomy by preserving the ileocecal valve. The rectum was transected at mid-sacral level. A patch of cecum with ileocecal valve was transected based on ileocolic artery. Twenty centimeters of distal ileum designed as S shape patch by suturing anti-mesenteric sides (figure 1). Then, ileocecal patch anastomosed to rectum in one layer using 4/0 vicril (figure 2), with long posterior rectal myotomy in hirschsprung cases.

The follow-up involved monthly visits for the first 6 months, visits every 3 months for the remaining 6 months, and then yearly visits. Sigmoidoscopy was done on all patients with ulcerative colitis and for the patients with familial polyposis yearly; biopsies were carried out to detect dysplastic changes in patients with ulcerative colitis and any evidence of new polyp configuration or malignant deterioration in familial polyposis cases. Clinical data as well as age at procedure, sex; primary disease, diagnostic modalities, and operative details were collected. Early and late functional consequences included (grow development, stool frequency, fecal soiling or incontinence and enterocolitis) were assessed through follow-up.

## Results

**Figure3:**
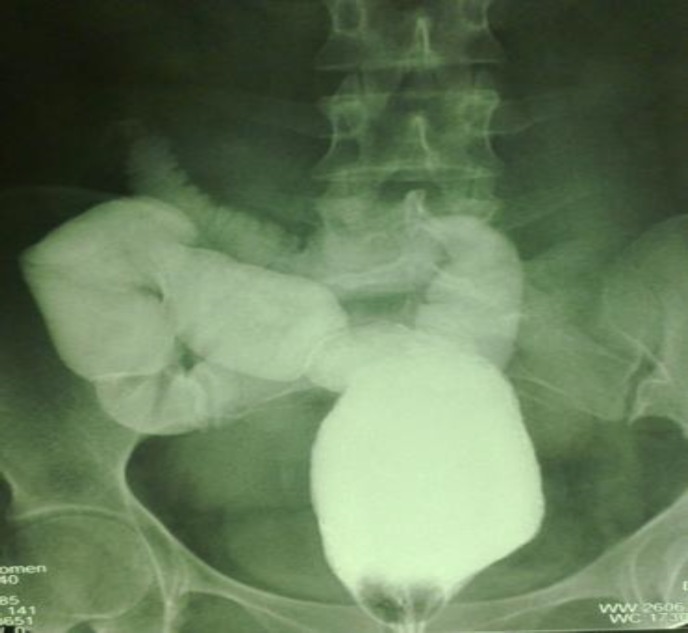
Barium enema study in a patient with ulcerative colitis 10 years after primary surgery

The mean age of the patients at the time of operation was 54 months (ranged from 2 months to 18 years). The mean age of the children with Hirschsprung’s disease at the time of operation was 11 months and the mean age of the children and adult with ulcerative colitis and familial polyposis was 13 years. Two cases of UC were siblings. There were 4 female and 10 male patients. The follow-up has ranged from 2 to 24 years (mean=11 years). There has been no death in the series. The patients were followed up annually. Clinical and endoscopic examinations or barium enema study (figure 3) was performed to evaluate the ileocecorectal anastomosis.

The main postoperative complications included recurrent enterocolitis in 2 cases with hirschsprung disease that responded to conservative management including bowel rest and cotrimoxazole and metronidazol therapy and rectal enema with normal saline. We had 2 cases of perianal fistula, one in a patient with ulcerative colitis and another in hirschsprung patient. Both of them led to fistulotomy and Seton insertion. Only 2 patients still are suffering from some degree of fecal soiling. There were 5 cases of adhesive bowel obstruction; one of them required an enterolysis in hirschsprung disease. Minor wound infections occurred in 2 patients. There were no anastomotic leaks, no cases of pouchitis encountered. 

In the first year, the patients experienced between four and six bowel movements during the day and one at night. Mean stool frequency 3 years after surgery was 3 per 24 hours. Seven cases with follow up period more than 5 years had only one bowel movement in 24 hours. Daytime continence was achieved in all patients one year after surgery except in one case with hirschsprung disease, that had rectal prolapsed and underwent Thiersch procedure, but was unsuccessful and soiling has continued up to now (17 years follow up). Annual rectosigmoidoscopy and mucosal biopsy revealed no dysplasia in ulcerative colitis patients and no polyps in FAP case.

## Discussion

We have 5 valves in GI tract (pharyngeal, cardia, pylorus, Ileocecal and rectum) that preserve them, help to improve the function of intestinal transit. The ileocecal valve is a papillose structure with physiological sphincter muscle (6). It is the only site in the GI tract for vitamin B12 and bile acid absorption. The authors have had great effort to achieve a perfect operation for benign disease of colorectal that removes the illness and save anorectal continence .There are many situations in children that need total proctocolectomy, but in all of them, ileocecal valve and rectum are sacrificed.

Most centers originally employ the straight pull-through technique for patients with ulcerative colitis and familial polyposis or Hirschsprung’s disease. Coran AG(2) in 1981, used endorectal pull-through for the management of ulcerative colitis in 26 children and adults. An abdominoperineal approach was used and mucosa and sub-mucosa removed from rectum and rectal tube were saved. He reported one case with rectal cuff abscess, and the median frequency of majority of them was seven per 24 hour. Most patients required the alternating use of Lomotil during first 6 to 12 months after operation. Displeasure with high stool frequencies and a major number of complications were encouraged to invent a variety of reservoirs to augment rectal capacitance and diminish stool frequency (7). Susan Galandiuk (8) between 1981 until 1989 studied 114 patients with pouch-related complications after IPAA that underwent reoperation. They divided pouch related complications into four groups: anastomotic stricture (n=42), perianal problems such as perianal abscess, fistula or sinuses (n=30), intraabdominal abscess or fistula (n=29), and functional pouch difficulties such as pouch empting problems or incontinence (n=13). Such as this study, there are many problems after IPAA. There are different surgical strategies in the management of FAP and ulcerative colitis. Although total proctocolectomy is an acceptable option in the reduction of colorectal cancer in them, but some others recommend to preserve the rectum for better protection pelvic innervations but risk of cancer is steady (1). On the other hand, some advocate proctectomy. In Von Roon AC etal’s (9) study in 2008, 78 patients with FAP who underwent colostomy and ileocecal anastomosis converted to protectomy and ileal pouch anastomosis and compared with primary IPAA. Pouch outcomes were similar except wound infection was more frequent in secondary IPAA. There are many studies for laparoscopic colectomy and ileal-pouch anal anastomosis. In most of them, post-operative outcomes are similar to open surgery (3, 10). Heise CP (4) in 2008 used laparoscopic restorative proctocolectomy as S-pouch design for surgical management of UC and FAP patients. But in all of them, duration of follow-up was short. Regarding to significant side effects with ileal pouch, some researchers preserved rectum in FAP patients for better quality of life in these patients but some of them reported no difference between the two groups (11). But our experience with preserved rectum and ileocecal valve is quite different than the skills accounted by other centers, with a stool frequency equivalent to that seen with reservoirs and an incidence of complications lower than that were reported with different pouches.

Total colonic agangliosis still represents an important problem in pediatric surgery. The authors use many procedures such as Martin which modified the Duhamel pull-through by using a longer segment of aganglionic rectum and colon to facilitate the absorption of water and electrolytes (12). He also preserved the entire colon and aganglionic colon underwent anastomosis to ganglionated small bowel, but results were poor (13) Kimura et al. (14) in 1988 advocated cecal patch for total colonic agangliosis. He reported this cecal patch provided an absorptive function equivalent to aganglionic rectum with excellent clinical results. Seiichi Goto and Grosfeld (15) in 1987 evaluated the efficacy of preserving the entire aganglionic colon with an antimesentric aganglionic colon patch in rates. When the entire aganglionic colon is preserved, rates showed severe abdominal distention and persistent diarrhea but segmental aganglionic intestinal patches delay transit time and increase the ability of fluid and electrolyte absorption.

The Rehbein-procedure (16) for children with hirschsprung disease (HD) is one of the current procedures used in Europe for the past 25 years. Reina Visser reported excellent long-term outcome in Rehbein despite a relatively large aganglionotic portion kept in situ compared with the other options such as the Soave, Swenson, or Duhamel procedures (17). The long-term results showed slightly more constipation after RB but less soiling compared with other techniques. In Rehbein –procedures, the length of the remained rectum is longer than our technique, so the risk of constipation is more. 

The recently published state’s pull-through as total aganglionosis that keep longer segment of rectum and anastomosed the ileum to rectum with posterior myotomy of rectum, this technique facilitate the pull-through operation (18). Although staged Duhamel procedure is now the selected procedure for the management of right-sided and total colonic Hirschsprung’s disease in most centers (19), but it needs facilities such as stapler that limits this procedure in some conditions. In all mentioned techniques, the authors have resected ileocecal valve, rectum or both for total colectomy in total colonic agangliosis, ulcerative colitis or FAP. Some used straight pull-through and the others used reservoir. Controversies are about the best procedure for anal anastomosis (either straight or pouch type), especially for total colonic agangliosis (20). But in our study, we used a novel way that preserved ileocecal valve and half of the rectum with S-shape ileum for prolonging intestinal transit. In long-term follow-up majority of them had one bowel movement a day and the risk of entrocolitis, stricture and incontinence were very low. 

In conclusion Ileocecal patch in total colectomy has the advantage of fecal continence with acceptable functional results; this may be considered the preferred operation for most patients with benign colorectal disease.

## Notes

**Funding:** This study was financially supported by the office of the Vice Chancellor, for Clinical Research of Tehran University of Medical Sciences.
